# High prevalence of obesity, central obesity and abnormal glucose tolerance in the middle-aged Finnish population

**DOI:** 10.1186/1471-2458-8-423

**Published:** 2008-12-29

**Authors:** Timo E Saaristo, Noël C Barengo, Eeva Korpi-Hyövälti, Heikki Oksa, Hannu Puolijoki, Juha T Saltevo, Mauno Vanhala, Jouko Sundvall, Liisa Saarikoski, Markku Peltonen, Jaakko Tuomilehto

**Affiliations:** 1 Finnish Diabetes Association, Kirjoniementie 15, FIN-33680, Tampere, Finland; 2 Department of Public Health, University of Helsinki, Finland; 3 Unit of Epidemiology and Clinical Research, University Hospital la Paz, Madrid, Spain; 4 Department of Internal Medicine, South Ostrobothnia Central Hospital, Finland; 5 Tampere University Hospital, Tampere, Finland; 6 Central Hospital of Middle Finland, Jyväskylä, Finland; 7 Laukaa Health Center, Finland; 8 National Public Health Institute, Helsinki, Finland

## Abstract

**Background:**

There is a worldwide increase in the prevalence of obesity and disturbances in glucose metabolism. The aim of this study was to assess the current prevalence of obesity, central obesity and abnormal glucose tolerance in Finnish population, and to investigate the associations between body mass index (BMI), waist circumference and abnormal glucose tolerance.

**Methods:**

A cross-sectional population-based survey was conducted in Finland during October 2004 and January 2005. A total of 4500 randomly selected individuals aged 45–74 years were invited to a health examination that included an oral glucose tolerance test. The participation rate was 62% in men and 67% in women.

**Results:**

The prevalence of obesity was 23.5% (95% Confidence Interval (CI) 21.1–25.9) in men, and 28.0% (95% CI 25.5–30.5) in women. The overall prevalence of abnormal glucose tolerance (including type 2 diabetes, impaired glucose tolerance, or impaired fasting glucose) was 42.0% (95% CI 39.2–44.8) in men and 33.4% (95% CI 30.9–36.0) in women. The prevalence of previously unknown, screen-detected type 2 diabetes was 9.3% (95% CI 7.7–11.0) in men and 7.3% (95% CI 5.9–8.7) in women. Central obesity was associated with abnormal glucose tolerance within each of the three BMI categories normal (< 25 kg/m^2^), overweight (25–29 kg/m^2^), and obese (≥ 30 kg/m^2^).

**Conclusion:**

In a population-based random sample of Finnish population, prevalences of obesity, central obesity and abnormal glucose tolerance were found to be high. A remarkably high number of previously undetected cases of type 2 diabetes was detected. Waist circumference is a predictor of abnormal glucose tolerance in all categories of obesity.

## Background

The increasing prevalence of obesity and sedentary lifestyle are the major underlying causes for type 2 diabetes (T2D) to become one of the fastest growing public health problems worldwide, imposing a high financial burden on health care costs [1,2]. The World Health Organization (WHO) has estimated that the number of adults with diabetes will more than double from an estimated 143 million in 1997 to 300 million by 2025 [3].

The prevalence of T2D in the 45–64 years-old population in Finland has been found to be 10.2% in men and 7.4% in women in 1992 [4]. The study conducted in Finland observed that there was a large number of clinically undiagnosed T2D cases [4]. This finding together with the data from the Diabetes Epidemiology – Collaborative analysis Of Diagnostic Criteria in Europe (DECODE) study based on earlier surveys [5] give reason to assume that the correct prevalence of diabetes in the population is markedly higher than recognized.

Abnormal glucose tolerance such as impaired glucose tolerance (IGT) and impaired fasting glucose (IFG) are modifiable risk factors of T2D and cardiovascular diseases [6,7]. Several studies have shown that the risk of developing abnormal glucose tolerance including T2D is closely linked to the presence and duration of overweight and obesity [8-10]. Indeed, 90% of individuals with T2D are either overweight or obese [3]. Furthermore, there is sufficient evidence from several studies to conclude that lifestyle intervention in people with IGT can result in sustained lifestyle changes and a reduction in diabetes incidence [11-16].

Given the current knowledge that visceral fat is metabolically more dangerous than subcutaneous fat, it is surprising that there is only limited information available about the association between the risk of abnormal glucose tolerance among overweight and obese people with increasing waist circumference [17-19].

The aims of this study were to investigate the current prevalence of obesity, central obesity and abnormal glucose tolerance as well as the associations between body mass index (BMI), waist circumference and abnormal glucose tolerance among 45–74 year-old Finnish men and women, and to assess whether the prevalence of abnormal glucose tolerance is associated with increased central obesity among people who are normal weight, overweight or obese.

## Methods

The FIN-D2D survey was carried out in hospital districts of Pirkanmaa, South Ostrobothnia and Central Finland during October 2004 and January 2005. A random sample of 4500 subjects aged 45–74 years, stratified according to sex, 10-year age groups (45–54, 55–64, and 65–74 years) and the three geographical areas, was selected from the National Population Register in September 2004. The study participants were invited to a clinical examination by mail. Together with the invitation, they also received a self-administered questionnaire on socioeconomic background, medical history, and health behavior. They were asked to complete the questionnaire at home, and bring it to the health examination.

Health examination was carried out according to the WHO MONICA project protocol [20]. Height, weight and waist circumference were measured by nurses specially trained for the survey procedures. Height was measured to the nearest 0.1 cm. Weight was measured in light clothing. Waist circumference was measured to the nearest cm. The examination also included an oral glucose tolerance test (OGTT), which was carried out according to the WHO recommendations [21]. A 300 ml test solution contained 75 g anhydrous glucose and 1.6 g citric acid. The test started after overnight fasting, and the 2-hour blood sample was obtained 120 minutes after the ingestion of the solution. Fasting and 2-hour samples for the plasma glucose determination were drawn into fluoridated tubes and centrifuged within 30 minutes. Plasma glucose was determined with a hexokinase method (Thermo Fisher Scientific Oy, Vantaa, Finland). All assays were performed at Laboratory of Analytical Biochemistry in National Public Health Institute, Helsinki.

### Definitions

Glucose tolerance was classified according to the WHO 1999 criteria [21]. Individuals who reported that they have diabetes were not included in the OGTT. Individuals who reported that they had diabetes onset at the age of younger than 35 years, and were treated with insulin from the diagnosis of their disease were classified as having type 1 diabetes. They constituted approximately 5% of all individuals who reported having diabetes, and they were not included in the type 2 diabetes prevalence calculations as persons with disease.

Individuals who had not T2D, and had fasting plasma glucose level ≥ 7.0 mmol/l or 2 h plasma glucose ≥ 11.1 mmol/l were classified as having screen-detected type 2 diabetes (ST2D). Those with 2 h plasma glucose ≥ 7.8 and < 11.1 mmol/l, and fasting plasma glucose < 7.0 mmol/l were classified as having IGT. IFG was defined as fasting plasma glucose ≥ 6.1 but < 7.0 mmol/l, and 2 h plasma glucose < 7.8 mmol/l. Subjects with T2D, ST2D, IGT or IFG were classified as having abnormal glucose tolerance.

BMI was calculated as weight (kg) divided by height^2 ^(m^2^). Central obesity was defined with the WHO criteria [22]: waist circumference ≥ 102 cm and ≥ 88 cm in men and women, respectively. In addition, central obesity in the International Diabetes Federation (IDF) definition of metabolic syndrome was also used: ≥ 94 cm in men and ≥ 80 cm in women [23].

The overall participation rates are given in the table 1. In addition to non-participants, individuals who were non-fasting at the time of blood sample or had other problems with OGTT were excluded from the analyses (Table 1). Totally, 1364 men and 1461 women were included in the analyses.

**Table 1:** Characteristics of the study participants according to sex and age groups.

	**MEN**				**WOMEN**			
	**45–54**	**55–64**	**65–74**	**Total**	**45–54**	**55–64**	**65–74**	**Total**
Number of subjects, invited	750	750	750	2250	750	750	750	2250
Participated (%)	417 (55.6)	497 (66.3)	482 (64.3)	1396 (62.0)	493 (65.7)	525 (70.0)	482 (64.3)	1500 (66.7)
Excluded from analysis*	12	12	8	32	14	14	11	39
Age (years)	50.2 (2.8)	59.6 (2.8)	69.5 (2.9)	60.3 (8.3)	50.2 (2.9)	59.5 (2.8)	70.0 (2.8)	59.8 (8.5)
Weight (kg)	86.2 (14.8)	86.8 (14.9)	83.5 (13.6)	85.8 (14.5)	72.3 (13.8)	73.4 (13.8)	72.6 (13.8)	72.7 (13.8)
BMI (kg/m^2^)	27.3 (4.2)	27.9 (4.2)	27.7 (4.0)	27.6 (4.1)	27.0 (5.0)	27.8 (5.1)	28.4 (5.2)	27.6 (5.2)
BMI < 25 kg/m^2^, %	31.1	23.3	24.5	26.8	40.5	34.4	26.3	34.7
BMI 25–29 kg/m^2^, %	48.1	51.5	50.4	49.7	35.9	36.6	40.6	37.3
BMI ≥ 30 kg/m^2^, %	20.7	25.2	25.1	23.5	23.6	29.0	33.1	28.0
Waist (cm)	97.3 (11.9)	100.3 (12.0)	99.9 (11.4)	99.3 (11.8)	87.6 (12.9)	90.3 (13.3)	91.5 (13.7)	89.6 (13.4)
Central obesity, WHO^#^, %	30.6	38.6	39.9	37.4	43.4	52.6	59.0	50.9
Central obesity, IDF^§^, %	59.8	71.3	73.6	68.2	71.2	75.7	81.1	75.0

Data are presented as means (standard deviation) except where noted otherwise.

*Number of subjects excluded from the analyses due to following problems with OGTT: refused to participate in OGTT (n = 3); vomiting during OGTT (n = 3); fasting duration unknown or less than < 8 hours (n = 12); time for OGTT deviated by more than 15 minutes from the 2 hour limit (n = 15); other problems with the blood sample (n = 15).

^#^Central obesity, WHO: waist circumference ≥ 102 cm in men and ≥ 88 cm in women.

^§^Central obesity in the IDF definition of metabolic syndrome: waist circumference ≥ 94 cm in men and ≥ 80 cm in women.

Total is an estimate of the population prevalence in the age group 45–74 years, taking into account the stratified sampling used in the study.

### Ethical considerations

The study protocol was approved by the research ethics committee of The Hospital District of Helsinki and Uusimaa. All the participants gave their written informed consent prior participation to the study.

### Statistical methods

Differences in glucose regulation by age groups, obesity and central obesity were compared with logistic regression models, using likelihood-ratio tests. Odds ratios for abnormal glucose regulation were calculated using the lowest obesity or central obesity category as the reference group. Analyses were done separately in both sexes, and adjusted for age and geographical area of living. The estimates of total prevalences in the age group 45–74 years were calculated taking into account the stratified sampling design used in the study. In addition, people were categorized jointly by BMI and waist circumference, and the proportion of participants with abnormal glucose regulation was assessed in each category, adjusting for age and sex. Analyses were performed with the statistics package Stata version 9.2 [24].

## Results

Table 1 shows the baseline characteristics of the study participants separately according to sex and 10-years age groups (Table 1). The overall response rate was 62% in men and 67% in women, respectively. The mean BMI was 27.6 in both men and women. The prevalence of obesity was 23.5% (95% CI 21.1–25.9) in men, and 28.0% (95% CI 25.5–30.5) in women. Central obesity increased with age in both men and women.

The total prevalence of T2D increased from 8.6% in the youngest age group to 24.9% in the oldest age group in men, and from 4.8% to 20.4% in women (Table 2). ST2D accounted for 57% in men and 65% in women of all prevalent cases of T2D. IGT and total abnormal glucose tolerance, including T2D, increased in both men and women with increasing age. Overall, abnormal glucose tolerance was observed in 42% of men and in 33% of women.

**Table 2:** Prevalence (95% confidence interval, CI) of type 2 diabetes (T2D), screen-detected type 2 diabetes (ST2D), total type 2 diabetes (TT2D), impaired glucose tolerance (IGT), impaired fasting glucose (IFG), abnormal glucose tolerance (AGT= TT2D, IGT, or IFG) in the study sample according to sex and age groups.

	**MEN**					**WOMEN**				
	**45–54**	**55–64**	**65–74**	**Total**	p*	**45–54**	**55–64**	**65–74**	**Total**	p*
**n**	405	485	474	1364		479	511	471	1461	
**T2D, %**	2.7	8.2	12.2	7.1	< 0.001	2.3	3.5	8.3	3.9	< 0.001
**(95% CI)**	(1.4–4.8)	(6.0–11.1)	(9.4–15.5)	(5.7–8.5)		(1.2–4.1)	(2.1–5.5)	(6.0–11.1)	(2.9–4.9)	
**ST2D, %**	5.9	8.7	12.7	9.3	0.002	2.5	8.0	12.1	7.3	< 0.001
**(95% CI)**	(3.8–8.7)	(6.3–11.5)	(9.8–16.0)	(7.7–11.0)		(1.3–4.3)	(5.8–10.7)	(9.3–15.4)	(5.9–8.7)	
**TT2D, %**	8.6	16.9	24.9	16.4	< 0.001	4.8	11.5	20.4	11.2	< 0.001
**(95% CI)**	(6.1–11.8)	(13.7–20.5)	(21.1–29.0)	(14.3–18.5)		(3.1–7.1)	(8.9–14.6)	(16.8–24.3)	(9.6–12.8)	
**IGT, %**	7.9	15.1	23.8	15.5	< 0.001	10.4	15.1	25.1	17.0	< 0.001
**(95% CI)**	(5.5–11.0)	(12.0–18.5)	(20.1–27.9)	(13.5–17.6)		(7.8–13.5)	(12.1–18.5)	(21.2–29.2)	(15.0–19.1)	
**IFG, %**	9.9	12.4	5.9	10.0	0.002	5.2	5.1	4.2	5.2	0.746
**(95% CI)**	(7.2–13.2)	(9.6–15.6)	(4.0–8.4)	(8.2–11.8)		(3.4–7.6)	(3.4–7.4)	(2.6–6.5)	(3.9–6.5)	
**AGT, %**	26.4	44.3	54.6	42.0	< 0.001	20.5	31.7	49.7	33.4	< 0.001
**(95% CI)**	(22.2–31.0)	(39.9–48.9)	(50.0–59.2)	(39.2–44.8)		(16.9–24.4)	(27.7–35.9)	(45.1–54.3)	(30.9–36.0)	

*p-values are for test of equivalence (likelihood-ratio test) between the three age-groups

Total is an estimate of the population prevalence in the age group 45–74 years, taking into account the stratified sampling used in the study.

Of all individuals who were categorized as having ST2D, 55% were identified with elevated 2 h plasma glucose values only, 17% with elevated fasting glucose values only, and 28% had both fasting and 2 h glucose values elevated. The importance of the OGTT to identify diabetes increased with increasing age; in the age group 45–54 years 42% of screen-detected diabetic cases were identified with the OGTT only, compared with 63% in the age group 65–74 years.

Men and women classified as obese (BMI = 30 kg/m^2^) had a 5-fold increased risk for T2D compared with normal weight people (Table 3). Odds ratios for T2D were 1.3 and 2.7 in overweight (BMI 25–29 kg/m^2^) men and women, respectively, as compared to those with normal weight. In addition, overweight and obese men and women had a significantly higher prevalence of IGT and IFG compared with normal weight people. Compared with normal weight individuals, odds ratios for abnormal glucose tolerance were 1.8 and 2.3 for overweight men and women, and 6.9 and 5.7 in obese men and women, respectively.

**Table 3:** Prevalence and odds-ratios (OR) with 95% confidence intervals of previously known type 2 diabetes (T2D), screen-detected type 2 diabetes (ST2D), total type 2 diabetes (TT2D), impaired glucose tolerance (IGT), impaired fasting glucose (IFG), abnormal glucose tolerance (AGT = TT2D, IGT or IFG) of the study sample according to BMI category and gender.

	**MEN**				**WOMEN**			
	**BMI**				**BMI**			
	**< 25**	**25–29**	**≥ 30**	p*	**< 25**	**25–29**	**≥ 30**	p*

**n**	355	684	325		494	550	417	
**T2D, %**	2.8	5.8	18.2	< 0.001	1.8	4.0	8.9	< 0.001
**OR†**	1.0 (ref)	2.05 (1.01–4.17)	7.59 (3.79–15.20)		1.0 (ref)	2.04 (0.92–4.49)	4.63 (2.20–9.77)	
**ST2D, %**	7.0	6.4	17.5	< 0.001	2.6	8.2	12.5	< 0.001
**OR**	1.0 (ref)	0.90 (0.54–1.50)	2.81 (1.70–4.65)		1.0 (ref)	2.92 (1.55–5.51)	4.60 (2.45–8.62)	
**TT2D, %**	9.9	12.3	35.7	< 0.001	4.5	12.2	21.3	< 0.001
**OR**	1.0 (ref)	1.25 (0.82–1.91)	5.27 (3.43–8.07)		1.0 (ref)	2.67 (1.61–4.41)	5.16 (3.15–8.45)	
**IGT, %**	10.4	17.1	19.7	0.004	10.1	16.9	24.5	< 0.001
**OR**	1.0 (ref)	1.78 (1.19–2.65)	2.06 (1.32–3.22)		1.0 (ref)	1.63 (1.12–2.38)	2.58 (1.77–3.75)	
**IFG, %**	5.9	9.2	13.5	0.002	2.0	4.5	8.6	< 0.001
**OR**	1.0 (ref)	1.65 (0.99–2.76)	2.63 (1.52–4.54)		1.0 (ref)	2.38 (1.13–5.02)	4.79 (2.33–9.83)	
**AGT, %**	26.2	38.6	68.9	< 0.001	16.6	33.6	54.4	< 0.001
**OR**	1.0 (ref)	1.80 (1.34–2.42)	6.94 (4.88–9.85)		1.0 (ref)	2.34 (1.72–3.17)	5.69 (4.15–7.80)	

*Likelihood-ratio test of equality, adjusted for age. †Age and geographical area adjusted odds ratios with 95% confidence intervals.

Central obesity, measured by large waist circumference, was associated with abnormal glucose tolerance within each of the three BMI categories normal (< 25 kg/m^2^), overweight, and obese (Table 4). The larger the waist circumference was, the higher the prevalence of any category of abnormal glucose tolerance.

**Table 4:** Prevalence and odds ratios (OR) with 95% confidence intervals of previously known type 2 diabetes (T2D), screen-detected type 2 diabetes (ST2D), total type 2 diabetes (TT2D), impaired glucose tolerance (IGT), impaired fasting glucose (IFG), abnormal glucose tolerance (AGT = TT2D, IGT or IFG) of the study sample by waist circumference and gender

	**MEN**				**WOMEN**			
	**WAIST CIRCUMFERENCE (cm)**				**WAIST CIRCUMFERENCE (cm)**			
	**< 94**	**94–101**	**≥ 102**	p*	**< 80**	**80–87**	**≥ 88**	p*

**n**	427	437	500		351	355	755	
**T2D, %**	2.1	5.5	15.2	< 0.001	2.0	2.5	6.9	< 0.001
**OR**†	1.0 (ref)	2.50 (1.14–5.47)	7.92 (3.89–16.11)		1.0 (ref)	1.20 (0.44–3.28)	3.12 (1.39–6.98)	
**ST2D, %**	5.2	7.8	14.0	< 0.001	2.3	3.7	11.8	< 0.001
**OR**	1.0 (ref)	1.46 (0.83–2.55)	2.69 (1.63–4.46)		1.0 (ref)	1.56 (0.63–3.82)	5.00 (2.39–10.49)	
**TT2D, %**	7.3	13.3	29.2	< 0.001	4.3	6.2	18.7	< 0.001
**OR**	1.0 (ref)	1.82 (1.14–2.89)	4.93 (3.24–7.51)		1.0 (ref)	1.40 (0.71–2.77)	4.48 (2.57–7.80)	
**IGT, %**	9.6	18.1	19.6	< 0.001	8.8	15.2	21.2	< 0.001
**OR**	1.0 (ref)	1.92 (1.28–2.90)	2.01 (1.35–3.00)		1.0 (ref)	1.82 (1.13–2.93)	2.50 (1.65–3.79)	
**IFG, %**	6.3	8.0	13.2	< 0.001	0.9	4.8	6.8	< 0.001
**OR**	1.0 (ref)	1.36 (0.80–2.29)	2.46 (1.53–3.96)		1.0 (ref)	5.97 (1.73–20.57)	8.71 (2.69–28.20)	
**AGT, %**	23.2	39.4	62.0	< 0.001	14.0	26.2	46.6	< 0.001
**OR**	1.0 (ref)	2.05 (1.51–2.79)	5.23 (3.87–7.05)		1.0 (ref)	2.18 (1.47–3.23)	5.02 (3.56–7.08)	

*Likelihood-ratio test of equality, adjusted for age. †Age and geographical area adjusted odds ratios including 95% confidence interval of odds ratios.

Table 5 shows the prevalence of ST2D, IGT, IFG and abnormal glucose tolerance for different waist circumference groups within the three BMI categories (Table 5). Among overweight study participants, the prevalence of abnormal glucose tolerance increased from 16% to 37% among the three waist circumference categories (p < 0.001).

**Table 5:** Age- and sex-adjusted prevalence of screen-detected T2D, IGT, IFG and AGT by BMI and waist circumference categories.

**MEN AND WOMEN**		**BMI**		
	**Waist:**	**< 25% (n)**	**25–29% (n)**	**≥ 30% (n)**

**ST2D**	**< 94/< 80**	3.7 (600)	3.0 (161)	0 (1)
	**94–101/80–87**	5.6 (197)	5.1 (532)	5.6 (30)
	**≥ 102/≥ 88**	8.1 (33)	9.2 (479)	15.9 (615)
	**p**	0.065	0.010	0.097
				
**IGT**	**< 94/< 80**	9.3 (600)	8.9 (161)	0 (1)
	**94–101/80–87**	12.2 (197)	17.7 (532)	16.0 (30)
	**≥ 102/≥ 88**	12.1 (33)	17.3 (479)	24.1 (615)
	**p**	0.387	0.042	0.249
				
**IFG**	**< 94/< 80**	3.2 (600)	3.6 (161)	0 (1)
	**94–101/80–87**	5.1 (197)	6.4 (532)	2.6 (30)
	**≥ 102/≥ 88**	0 (33)	8.1 (479)	12.9 (615)
	**p**	0.569	0.032	0.099
				
**AGT**	**< 94/< 80**	16.7 (600)	15.5 (161)	0 (1)
	**94–101/80–87**	24.2 (197)	30.1 (532)	24.3 (30)
	**≥ 102/≥ 88**	23.0 (33)	36.6 (479)	56.5 (615)
	**p**	0.045	< 0.001	0.001

Excluding people with known diabetes from the calculations.

p: test of trend within the BMI category, adjusted for age and sex.

## Discussion

In spite of raising concern about the global diabetes epidemic there are actually surprisingly few up to date reports published in various countries on the size of the current problem based on repeated cross-sectional population-based surveys. Such surveys are the only way to assess the situation at the population level and to monitor possible changes in prevalence of T2D and other abnormalities in glucose regulation.

This study assessed the prevalence of different categories of abnormal glucose tolerance at baseline of the implementation of the Finnish T2D prevention programme in three geographical areas in 2004 and 2005. In the survey, a large number of previously undetected diabetes cases were identified. As expected, both obesity and central obesity were related to abnormal glucose tolerance. Importantly, the risk of abnormal glucose tolerance increased with increasing central obesity within each of the obesity categories.

The overall prevalence of screen-detected diabetes of 9.3% and abnormal glucose tolerance of 42.0% in men, and 7.3% and 33.4% in women, respectively, are all high. The prevalence increased steadily with age, except that of IFG. Our results are in line with the data from previous surveys that many cases of diabetes remain undetected in the population since no systematic screening using an OGTT is conducted [25-28]. Moreover, the proportion on previously undiagnosed cases of diabetes seems to be larger than found in previous studies [29]. There seems to be sufficient evidence from various countries in the world to conclude that the undetected diabetes cases account for 30–60% of all diabetes cases. It is known that T2D often starts slowly, virtually without typical symptoms of diabetes. Thus, it is obvious that many cases of T2D remain undiagnosed for years. Moreover, if only fasting glucose measurements are used to screen for T2D, a significant proportion of asymptomatic cases of diabetes remain undetected, as also demonstrated by the DECODE study [30].

Most previous studies have demonstrated an association between development of glucose intolerance and increased BMI [27,31-33] and waist circumference [25]. The Cooperative Health Research in the Region of Augsburg (KORA) survey conducted in 2000, showed that both BMI and waist circumference were significantly higher in men and women with abnormal glucose tolerance [25]. People with undetected diabetes had a higher risk of being obese or having central obesity. The odds ratios in men and women with undiagnosed diabetes mellitus compared to non-diabetic subjects for obesity were 1.9 and those for central obesity -1.9 in women and 2.6 in men. Zargar et al. reported a 2.3-fold increase in risk of abnormal glucose tolerance in Asian Indian people aged 40 years or over with BMI > 25 kg/m^2 ^[28]. A waist-to-hip ratio greater than 1 doubled the risk of abnormal glucose tolerance.

Of all subjects who were categorized as ST2D, more than half were identified with elevated 2 h plasma glucose values alone having normal fasting glucose values. Thus, the findings of our study provide further scientific evidence that the measurement of fasting glucose alone can not be considered as the sole measurement in detecting abnormal glucose regulation in non-diabetic subjects [5].

Surprisingly, there is only limited information available about the prevalence of diabetes according to waist circumference within the BMI categories [18,19], [33,34]. Our results suggest that there seem to be difference in risk of diabetes even within obese and overweight people according to their waist circumference. Thus, reducing central obesity while BMI remains stable may lower the risk of diabetes.

T2D is characterized by a long preclinical phase including early stages of glucose metabolism disorders such as IGT and IFG [35]. Due to the long period without clinical symptoms, up to 50% of the patients are not aware of their disease [27,36,37] and macrovascular lesions are observed in many diabetic patients already at the time of diagnosis [26,38]. Several studies provided evidence that lifestyle intervention in people with abnormal glucose tolerance can result in lifestyle changes which lead to a reduction in diabetes incidence [11-15]. In the extended follow-up of the Finnish Diabetes Prevention Study (DPS), beneficial lifestyle changes and the corresponding reduction in diabetes incidence were maintained for several years after the individual lifestyle counseling was stopped [16]. Therefore, screening for undetected cases of T2D and implementing primary prevention measures among high risk individuals (e.g. subjects with IGT or IFG) might provide an optimal strategy to prevent many late complications of diabetes on long-term. The results from the Atherosclerosis Risk in Communities Study suggested that screening strategies based on fasting plasma glucose, complemented by clinical detection rules and/or an OGTT, are effective and practical in the detection of hyperglycemic states meriting clinical intervention [39]. Moreover, the KORA survey calculated that the number needed to screen (NNS) for undiagnosed diabetes was 6.5 for obese men and 9.7 for obese women, respectively 5.1 for men with central obesity (> 109 cm) and 8.5 for women with central obesity (> 100 cm) [25]. On the other hand, non-invasive and simple risk scores to detect undiagnosed diabetes or to predict the future probability of T2D have been developed, one of them in Finland [40]. The Finnish Diabetes Risk Score is now used in Finland nationwide as a part of the national prevention programme for T2D [41].

The strengths of this study are that it is based on a large representative sample of middle-aged men and women of three hospital districts in Finland using oral glucose tolerance tests to detect glucose metabolism disorders. However, several limitations of this study need to be considered. Despite efforts to increase the response rate, only 56% of the men in the age-group 45 to 54 years agreed to participate. The response rate was higher in women and among older men. Non-participants might have had already diabetes, and thus they have considered unnecessary to take part in the survey. Therefore, the prevalence of known type 2 diabetes might have been underestimated. The reason for the low participation in younger age groups may be due to the time constraint since the survey requires an attendance of at least two hours which may be difficult to arrange for working people during the day-time.

## Conclusion

Testing for diabetes and impaired glucose regulation using an OGTT in a population-based random sample detected a remarkably high number of previously undetected cases of type 2 diabetes in the Finnish population. This study adds more evidence to justify opportunistic diabetes screening in subjects with known cardiovascular risk factors or history of cardiovascular disease as recently emphasized. The main modifiable risk factors such as obesity and central obesity were significantly related to a higher prevalence of abnormal glucose tolerance in Finland. Furthermore, the high prevalence of central obesity and obesity among middle-aged Finnish people stresses the importance and urgent implementation of strategies for weight management in the general population in order to decrease abnormal glucose tolerance, and in particular the onset of diabetes. Currently a national diabetes prevention programme is ongoing in Finland, and its effects will be evaluated by subsequent follow-up surveys in the same target population of this study.

## Competing interests

The authors declare that they have no competing interests.

## Authors' contributions

TES and NCB made substantial contributions to conception, design, and interpretation of the data and they involved in drafting the manuscript. All authors participated in the design and performing of the study, acquisition of data and helped to draft the manuscript or revising it. MP performed the statistical analysis. All authors read and approved the final manuscript.

## Pre-publication history

The pre-publication history for this paper can be accessed here:



## References

[B1] King H, Aubert RE, Herman WH (1998). Global burden of diabetes, 1995–2025: Prevalence, numerical estimates, and projections. Diabetes Care.

[B2] Wild S, Roglic G, Green A, Sicree R, King H (2004). Global prevalence of diabetes: Estimates for the year 2000 and projections for 2030. Diabetes Care.

[B3] Kumanyika S, Jeffery RW, Morabia A, Ritenbaugh C, Antipatis VJ (2002). Obesity prevention: the case for action. Public Health Approaches to the Prevention of Obesity (PHAPO) Working Group of the International Obesity Task Force (IOTF). Int J Obes Relat Metab Disord.

[B4] Ylihärsila H, Lindström J, Eriksson JG, Jousilahti P, Valle TT, Sundvall J, Tuomilehto J (2005). Prevalence of diabetes and impaired glucose regulation in 45- to 64-year-old individuals in three areas of Finland. Diabet Med.

[B5] The DECODE Study Group (2003). Age- and sex-specific prevalences of diabetes and impaired glucose regulation in 13 European cohorts. Diabetes Care.

[B6] Harris MI (1996). Impaired glucose tolerance: prevalence and conversion to NIDDM. Diabet Med.

[B7] Nathan DM, Davidson MB, DeFronzo RA, Heine RJ, Henry RR, Pratley R, Zinman B (2007). ADA Consensus Statement. Impaired fasting glucose and impaired glucose tolerance: Implications for care. Diabetes Care.

[B8] Colditz GA, Willett WC, Rotnitzky A, Manson JE (1995). Weight gain as a risk factor for clinical diabetes mellitus in women. Ann Intern Med.

[B9] Must A, Spadano J, Coakley EH, Field AE, Colditz G, Dietz WH (1999). The disease burden associated with overweight and obesity. JAMA.

[B10] Hu FB, Manson JE, Stampfer MJ, Colditz G, Liu S, Solomon CG, Willet WC (2001). Diet, lifestyle, and the risk of type 2 diabetes mellitus in women. N Engl J Med.

[B11] Pan XR, Li GW, Hu YH, Wang JX, Yang WY, An ZX, Hu ZX, Lin J, Xiao JZ, Cao HB, Liu PA, Jiang XG, Jiang YY, Wang JP, Zheng H, Zhang H, Bennet PH, Howard BV (1997). Effects of diet and exercise in preventing NIDDM in people with impaired glucose tolerance. The Da Qing IGT and diabetes study. Diabetes Care.

[B12] Tuomilehto J, Lindström J, Eriksson JG, Valle TT, Hämäläinen H, Ilanne-Parikka O, Keinänen-Kiukaanniemi S, Laakso M, Louheranta A, Rastas M, Salminen V, Uusitupa M (2001). Prevention of type 2 diabetes mellitus by changes in lifestyle among subjects with impaired glucose tolerance. N Engl J Med.

[B13] Diabetes Prevention Program Research Group (2002). Reduction in the incidence of type 2 diabetes with lifestyle intervention or metformin. N Engl J Med.

[B14] Ramachandran A, Snehalatha C, Mary S, Mukesh B, Bhaskar AD, Vijay V, Indian Diabetes Prevention Programme (IDPP) (2006). The Indian Diabetes Prevention Programme shows that lifestyle modification and metformin prevent type 2 diabetes in Asian Indian subjects with impaired glucose tolerance (IDPP-1). Diabetologia.

[B15] Kosaka K, Noda M, Kuzuya T (2005). Prevention of type 2 diabetes by lifestyle intervention: a Japanese trial in IGT males. Diabetes Res Clin Pract.

[B16] Lindstrom J, Ilanne-Parikka P, Peltonen M, Aunola S, Eriksson JG, Hemiö K, Hämäläinen, Härkönen P, Keinänen-Kiukaanniemi S, Laakso M, Louheranta A, Mannelin M, Paturi M, Sundvall J, Valle TT, Uusitupa M, Tuomilehto J (2006). Sustained reduction in the incidence of type 2 diabetes by lifestyle intervention: follow-up of the Finnish Diabetes Prevention Study. Lancet.

[B17] Kurioka S, Murakami Y, Nishiki M, Sohmiya M, Koshimura K, Kato Y (2002). Relationship between visceral fat accumulation and anti-lipolytic action of insulin in patients with type 2 diabetes mellitus. Endocr J.

[B18] Vanhala MJ, Pitkäjärvi TK, Kumpusalo EA, Takala JK (1998). Obesity type and clustering of insulin resistance-associated cardiovascular risk factors in middle-aged men and women. Int J Obes Relat Metab Disord.

[B19] Wang Y, Rimm EB, Stampfer MJ, Willet WC, Hu FB (2005). Comparison of abdominal adiposity and overall obesity in predicting risk of type 2 diabetes among men. Am J Clin Nutr.

[B20] WHO MONICA Project Principal Investigators (1988). The World Health Organization MONICA Project (monitoring trends and determinants in cardiovascular disease): a major international collaboration. J Clin Epidemiol.

[B21] WHO Consultation (1999). Definition, diagnosis and classification of diabetes mellitus and its complications. Part 1: diagnosis and classification of diabetes mellitus.

[B22] Report of WHO Consultation (2000). Obesity: Preventing and managing the global epidemic. Geneva, WHO Technical Report Series 894.

[B23] International Diabetes Federation (2005). IDF consensus worldwide definition of the metabolic syndrome. International Diabetes Federation.

[B24] StataCorp (2005). Stata Statistical Software. Release 90 College Station, TX: StataCorp LP.

[B25] Rathmann W, Haastert B, Icks A, Löwel H, Meisinger C, Holle R, Giani G (2003). High prevalence of undiagnosed diabetes mellitus in Southern Germany: target populations for efficient screening. The KORA survey 2000. Diabetologia.

[B26] Harris MI, Klein R, Welborn TA, Knuiman MW (1992). Onset of NIDDM occurs at least 4–7 yr before clinical diagnosis. Diabetes Care.

[B27] Gregg EW, Cadwell BL, Cheng YJ, Cowie CC, Williams DE, Geiss L, Engelgau MM, Vinicor F (2004). Trends in the prevalence and ratio of diagnosed to undiagnosed diabetes according to obesity levels in the U.S. Diabetes Care.

[B28] Zargar AH, Khan AK, Masoodi SR, Laway BA, Wani AI, Bashir MI, Dar FA (2000). Prevalence of type 2 diabetes mellitus and impaired glucose tolerance in the Kashmir Valley of the Indian subcontinent. Diabetes Res Clin Pract.

[B29] Valle T, Tuomilehto J, Eriksson J, Alberti KGMM (1997). Epidemiology of NIDDM in Europids. International Textbook of Diabetes Mellitus.

[B30] The DECODE Study Group (1999). Is fasting glucose sufficient to define diabetes? Epidemiological data from 20 European studies. Diabetologia.

[B31] Harris MI, Hadden WC, Knowler WC, Bennet PH (1987). Prevalence of diabetes and impaired glucose tolerance and plasma glucose levels in U.S. population aged 20–74 yr. Diabetes.

[B32] Omar MA, Seedat MA, Dyer RB, Motala AA, Knight LT, Becker PJ (1994). South African Indians show a high prevalence of NIDDM and bimodality in plasma glucose distribution patterns. Diabetes Care.

[B33] Janssen I, Heymsfield SB, Allison DB, Kotler DP, Ross R (2002). Body mass index and waist circumference independently contribute to the prediction of nonabdominal, abdominal subcutaneous and visceral fat. Am J Clin Nutr.

[B34] Collins VR, Dowse GK, Toelupe PM, Imo TT, Aloaina FL, Spark RA, Zimmet PZ (1994). Increasing prevalence of NIDDM in the Pacific Island population of Western Samoa over a 13-year period. Diabetes Care.

[B35] The DECODE Study Group on behalf of the European Diabetes Epidemiology Group (1999). Consequences of the new diagnostic criteria for diabetes in older men and women. DECODE Study (Diabetes Epidemiology: Collaborative Analysis of Diagnostic Criteria in Europe). Diabetes Care.

[B36] Mayor S (2005). Quarter of people with diabetes in England are undiagnosed. BMJ.

[B37] Koopman RJ, Mainous AG, Jeffcoat AS (2004). Moving from undiagnosed to diagnosed diabetes: the patient's perspective. Fam Med.

[B38] Kohner EM, Aldington SJ, Stratton IM, Manley SE, Holman RR, Matthews DR, Turner RC (1998). United Kingdom Prospective Diabetes Study, 30: diabetic retinopathy at diagnosis of non-insulin-dependent diabetes mellitus and associated risk factors. Arch Ophthalmol.

[B39] Schmidt MI, Duncan BB, Vigo A, Pankow J, Ballantyne CM, Couper D, Brancati F, Folsom AR, for the ARIC investigators (2003). Detection of undiagnosed diabetes and other hyperglycemia states: The Atherosclerosis Risk in Communities Study. Diabetes Care.

[B40] Lindström J, Tuomilehto J (2003). The diabetes risk score: a practical tool to predict type 2 diabetes risk. Diabetes Care.

[B41] Saaristo T, Peltonen M, Keinänen-Kiukaanniemi S, Vanhala M, Saltevo J, Niskanen L, Oksa H, Korpi-Hyövälti E, Tuomilehto J, for the FIN-D2D Study Group (2007). National type 2 diabetes prevention programme in Finland: FIN-D2D. International Journal of Circumpolar Health.

